# Adjustment Disorders Are Uniquely Suited for eHealth Interventions: Concept and Case Study

**DOI:** 10.2196/mental.4157

**Published:** 2015-05-08

**Authors:** Andreas Maercker, Rahel C Bachem, Louisa Lorenz, Christian T Moser, Thomas Berger

**Affiliations:** ^1^ Division of Psychopathology and Clinical Intervention Department of Psychology University of Zurich Zurich Switzerland; ^2^ Institute of Psychology Department of Clinical Psychology and Psychotherapy University of Berne Berne Switzerland

**Keywords:** adjustment disorders, intervention, e-mental health, unguided self-help, depression

## Abstract

**Background:**

Adjustment disorders (also known as mental distress in response to a stressor) are among the most frequently diagnosed mental disorders in psychiatry and clinical psychology worldwide. They are also commonly diagnosed in clients engaging in deliberate self-harm and in those consulting general practitioners. However, their reputation in research-oriented mental health remains weak since they are largely underresearched. This may change when the International Statistical Classification of Diseases-11 (ICD-11) by the World Health Organization is introduced, including a new conceptualization of adjustment disorders as a stress-response disorder with positively defined core symptoms.

**Objective:**

This paper provides an overview of evidence-based interventions for adjustment disorders.

**Methods:**

We reviewed the new ICD-11 concept of adjustment disorder and discuss the the rationale and case study of an unguided self-help protocol for burglary victims with adjustment disorder, and its possible implementation as an eHealth intervention.

**Results:**

Overall, the treatment with the self-help manual reduced symptoms of adjustment disorder, namely preoccupation and failure to adapt, as well as symptoms of depression, anxiety, and stress.

**Conclusions:**

E-mental health options are considered uniquely suited for offering early intervention after the experiences of stressful life events that potentially trigger adjustment disorders.

## Introduction

### E-Mental Health Interventions and Adjustment Disorder

E-mental health interventions have made significant progress in the treatment of common mental disorders. For all major groups of mental disorders, effective e-interventions are available, for example [1,2]. There is one diagnostic group, however, that received little attention in e-mental health, the adjustment disorders (also known as mental distress in response to a stressor). Studies scarcely included the adjustment disorder diagnosis in e-mental health or telemedicine contexts [3-5]. In population-based epidemiological studies, prevalence rates of adjustment disorder range from 0.5%-2% [6]. Comprehensive mental service utilization studies indicate that high proportions of clients have an adjustment disorder diagnosis, with up to 30% in general practice or consultant-liaison services [7]. Experts agree that the previous definition of adjustment disorders in the psychiatric classification systems is still problematic for various reasons [8]. However, the two most recent revisions of the Diagnostic and Statistical Manual of Mental Disorders, 5th Version (DSM-5) and the Beta-Version of the International Classification of Diseases 11 (ICD-11) provide several improvements with respect to the previous issues [7]. Consequently, it seems reasonable for the field of e-mental health to consider its potential contributions to the progress and satisfaction of clinical needs in the area of adjustment disorders.

The current paper is organized in two main parts: first, it provides an overview about general aspects of adjustment disorders, its distinction from other disorders, current diagnostic and treatment approaches, as well as the current empirical status; and second, it presents the outline of a new structured cognitive behavior therapy (CBT) program for treating adjustment disorders (sample case, burglary victims) and illustrates this by intervention results of a single case.

### Distinctiveness From Other Disorders

An adjustment disorder is defined as a transient maladaptive or pathological reaction to identifiable stressors or changes in life circumstances with symptoms emerging within three months of the onset of the stressor, according to DSM-5 and ICD-10. Adjustment disorders are triggered by serious, but nontraumatic, stressors, which usually are severe life events. The stressors can either be acute (eg, loss of work, break-up of a romantic relationship, trouble with a family member), chronic (eg, financial burden, family or occupational problems), recurring (eg, seasonal economic slumps), or continuous (eg, living in a criminal environment) [6]. Adjustment disorder symptom criteria include various types of behavioral symptoms, which are manifested far beyond the expected magnitude when confronted with such a burdensome event. In addition, the disorder leads to significant social, occupational, or academic performance-related impairments.

The distinction between adjustment disorder and a normal stress response is based on the severity and the duration of symptoms or impairments. The criterion of impact on personal functioning takes into account the nature of the stressor, the personal and interpersonal context in which it has occurred, and cultural norms with regard to such responses. A recent study in preparation of the new classification system ICD-11 shows sufficient discriminant validity for distinguishing nondisorders (ie, normal stress response) from adjustment disorder with more than 2500 practitioners across several countries [9].

In contrast, posttraumatic stress disorder (PTSD) and acute stress reaction require the presence of a stressor of a magnitude that would be traumatic for almost everybody, as well as a specific symptom constellation [10]. Since not everybody exposed to traumatic events responds by developing PTSD, but may nevertheless develop significant symptoms and/or functional impairment, adjustment disorder should be considered as an alternative diagnosis [7,11].

Distinguishing adjustment disorder from a depressive episode (DE) may be particularly challenging. The appearance of a DE is often linked to a precipitating severe life event. If all diagnostic requirements of a DE are fulfilled (low mood, loss of interests and energy for at least two weeks), often along with a known history of a previous depressive disorder, the diagnosis of depression is appropriate. Adjustment disorder differs from DE by the fact that it remits when the stressor is removed or a new level of adaptation is reached. Doherty et al [12] empirically distinguished adjustment disorder and DE in a liaison psychiatry sample by means of a precipitating events list, the Beck Depression Inventory (BDI), and a social support scale. They identified 5 criteria distinguishing between both diagnoses with excellent predictive values: (1) a relationship breakdown event (for adjustment disorder), (2) high BDI (for DE), versus (3) low BDI (for adjustment disorder), (4) higher self-blame (for adjustment disorder), and (5) higher self-reported social support (for adjustment disorder). Doherty et al [12] published a similar study, additionally including a personality measure. Fewer depressive symptoms, fewer problems with social contacts, and a less pronounced disposition toward perfectionism were predictors of adjustment disorder. It is expected more studies will soon appear in this area cf. [7].

### Recent Diagnostic Approaches, Diagnostic and Statistical Manual of Mental Disorders, 5th Version and International Classification of Diseases 11

Until now adjustment disorder symptom criteria are rather ill defined, for example, including various kinds of behavioral symptoms, which are manifested far beyond the expected magnitude when confronted with such a burdensome event. The lack of explicitly defined or pathognomonic symptoms had been widely criticized since it impedes empirical research [8].

To overcome these general deficits, it was decided to enhance the disorder’s characterization in the two common classification systems for mental disorders, DSM and ICD, by defining adjustment disorder as a stress-response disorder. This relates adjustment disorder to a useful biological context within the framework proposed by Selye [13] and to the key role of the hypothalamic-pituitary-adrenocortical (HPA) system in the human stress response. Such work has been updated by a current, more sophisticated understanding of the neurocircuitry and the psychobiological systems that mediate and moderate this response [14]. This understanding also provides a perspective for the development of therapeutic approaches that effectively produce clinical remission in PTSD, acute stress disorder, or prolonged grief disorder, and thus may inform new therapeutic innovations for adjustment disorder.

The current DSM-5 describes five criteria for the diagnosis of an adjustment disorder: (1) emotional or behavioral symptoms arising within 3 months of exposure to an identifiable stressor; (2) the symptoms must be clinically significant or exceeding what is expected in response to the stressor and/or existing significant impairment in social or occupational functioning; (3) the stress-related disturbance is not due to another mental disorder or merely exacerbating a preexistent mental disorder; (4) the symptoms do not represent normal bereavement; and (5) the symptoms do not persist for more than 6 months once the stressor or its consequence have terminated [15]. However, criticism has been raised regarding the failure of DSM-5 to provide specific diagnostic criteria and to guide clinicians in distinguishing problematic responses from normal adaptive reactions to stress [12].

The Beta-Version of ICD-11 introduces two core symptom groups for adjustment disorder, preoccupations with the stressor and failure to adapt [10]. Consistent with DSM-5, these symptoms have to arise within 3 months of exposure to an identifiable stressor or multiple stressors. The symptoms typically resolve within 6 months, unless the stressor persists for a longer period. Examples include divorce, illness or disability, socioeconomic problems, and conflicts at home or at work. The reaction to the stressor is characterized by preoccupation with the stressor or its consequences, including excessive worry, recurrent and distressing thoughts about the stressor, or constant rumination about its implications. Failure to adapt to the stressor causes significant impairment in personal, family, social, educational, occupational, or other important areas of functioning. If functioning is maintained only through significant additional effort, or is significantly impaired compared to the individual's prior functioning or what would be expected, he or she would be considered impaired due to the disturbance.

A previous version of the ICD-11 proposal had been empirically validated in different samples in epidemiological, etiological, and, initially, in treatment research [6]. Direct comparisons between the DSM-5 and ICD-11 Beta approach are still lacking. Nevertheless, both new classificatory approaches will stimulate further innovations and investigations for this underresearched area.

### Approaches to Adjustment Disorder Treatment

Despite high prevalence rates and the fact that adjustment disorder patients suffer from a significant decrease in quality of life and an increased risk of suicidal behavior [16], only few specific treatment approaches are available to date. This may partly be due to the paucity of explicit theoretical models of adjustment disorder on which intervention programs could be based. Additionally, the indistinctive diagnostic criteria of ICD-10 and DSM-IV may account for the lack of empirically validated interventions. However, two theoretical models are suitable to explain the development of an adjustment disorder.

In the “crisis model”, Caplan [17] postulated typical trajectories that occur after extreme stress and that destabilize the individual. A personal crisis is defined as a problem that is unsolvable for the individual concerned. Caplan proposed that psychopathological symptoms develop when an individual has insufficient or inflexible defense mechanisms to handle the problem. Such personal crises are comparable to critical life events that might cause adjustment disorder. A second useful model for psychotherapy of adjustment disorder is the stress-response model by Horowitz [18]. Within this model, adjustment disorder, along with PTSD and complicated grief disorder, were conceptualized as stress response syndrome for the first time to our knowledge [19]. The model proposes that a severe stressor evokes intensive negative emotions, which in a next step are reacted upon through suppression, avoidance, or dysfunctional behavior. Consequently, a vicious circle of intrusive memories and avoidance responses arises in the individual. This discontinues as soon as a phase of working-through takes place within a person and cognitive processing enables a decrease of preoccupation and maladaptive behaviors.

Regarding treatment, several authors recommended a modular approach including therapeutic elements from a variety of approaches specific for other disorders [20,21]. An individual etiological model, the analysis of competences and resources, and the integration of the critical life event are considered important parts in adjustment disorder therapy. Maercker [22] suggests to put a special focus on techniques adapted from PTSD treatment, such as exposition (eg, imaginative or narrative exposition, writing assignments), cognitive restructuring (eg, for recurrent distressing thoughts about the stressor, blaming oneself or others), and eye movement desensitization and reprocessing (EMDR).

### Empirical Evaluations

A limited amount of controlled clinical trials have been conducted in order to evaluate the different treatment approaches for adjustment disorder in a face-to-face context. They comprise a wide variety of therapeutic techniques depending on the clinical focus of the authors and range from problem solving training, grief work, and anxiety coping strategies to client-centered psychotherapy.

The first study, to our knowledge, to apply techniques from the treatment of PTSD was conducted by Cvetek [23], who introduced EMDR as a technique for reducing anxiety due to intrusions of stressful memories. Three hours of EMDR were compared with a placebo control condition (active listening for three hours) and a wait list condition. The EMDR group showed significantly lower anxiety scores and less intrusive and avoidance symptoms compared to the control groups.

In a randomized controlled design, Van der Klink et al [24] evaluated an adjustment disorder intervention aiming at the development of problem solving strategies for daily life problems including elements of time management, stress inoculation, and cognitive restructuring. Compared to treatment as usual, the intervention led to shorter sickness leave and lower recurrence rates, but showed no difference in psychopathological symptoms.

Taking a different theoretical approach, Altenhöfer et al [25] evaluated 12 sessions of client-centered psychotherapy in comparison to a wait list in a nonrandomized trial and found lasting treatment effects for adjustment disorder symptoms, life satisfaction, and general functioning. The effects were maintained in a 2 year catamnesis study [26]. Furthermore, brief dynamic therapy and brief supportive therapy were both applied in a sample of patients suffering from minor depression and adjustment disorder. Both approaches produced significant improvement in depression and anxiety compared to a nontreated control group [27]. In a later study by Ben-Itzhak et al [28], short-term psychodynamic treatment (3 months) was found to be as effective as intermediate dynamic therapy (12 months). The authors concluded that brief interventions seem thereby well suited for the treatment of adjustment disorder.

There are two intervention studies in a group setting that are available. First, a German cognitive-behavioral manual provides a modular approach to facilitate adjustment to significant life stressors in 10 sessions [21]. In a clinical trial, a significant decrease in anxiety, anger, and an increase in mood were achieved compared to the wait list condition. Second, a recent study by Hsiao et al [29] randomly assigned adjustment disorder patients to an eight week body-mind-spirit group psychotherapy and a control group (one session psychoeducation). The intervention focused on enhancing patients’ resilience to cope with stress. With regard to depression and anxiety symptoms, no differential change was achieved, however, suicidal ideation was significantly reduced and HPA axis hyperactivity was reduced in the intervention group.

It is generally agreed that psychotherapy is the treatment of choice in adjustment disorder and very few pharmacotherapy studies are available to date [20]. Surprisingly, a recent study found that 37% of the patients with adjustment disorder are prescribed a psychotropic drug [30].

In conclusion, there are several promising psychotherapeutic approaches that have shown to be effective in treating adjustment disorder in several empirical studies. However, these studies are based on very heterogeneous theoretical foundations and a need for replication of the results is apparent.

### E-Mental Health-Based Interventions

A vast amount of research in the area of e-mental health has been conducted during the past decade, particularly for the highly prevalent disorders such as depression and anxiety disorders, for example [31]. Common e-mental health interventions are based on CBT approaches and include unguided self-help treatments and guided self-help interventions with varying degrees of therapist contact [32]. E-interventions are effective approaches in the treatment of various psychological health problems, for example [2], and they have several advantages such as easy accessibility, easy use independent of time and place at a self-determined pace, and low cost of delivery to large populations [32].

As adjustment disorder is highly prevalent and frequently diagnosed by general practitioners, a large part of patients go without psychotherapeutic treatment [33]. Consequently, there is a need for widely available treatment options. E-mental health interventions have the capacity to meet this demand. In addition, the transient character of adjustment disorder makes it uniquely suited for low-threshold interventions.

### Previous E-Mental-Health-Based Approach

Until now, only one intervention in the domain of e-mental health was devoted to adjustment disorder [3] using the virtual reality (VR) program "EMMA's world" [34]. The authors developed this VR program aiming at the introduction of positive mood and joy. Furthermore, the virtual world enables patients to confront, accept, and handle difficult emotions and cognitions connected to a stressful life event. Strategies are deduced from positive psychology attempting to increase the natural ability of humans to resist and grow in adverse circumstances [35]. The VR program is meant to be used in combination with face-to-face elements, and comprises six weekly sessions. In every session, patients are introduced to new concepts by the therapist and then proceed to the emotional processing part of the "EMMA's world" program [34]. This program is used to activate and process emotions and cognitions associated with the stressful event and to provide exposure to anything that is avoided in order to allow processing of all emotional aspects of the event. The approach builds upon a rationale developed by Foa and Kozak [36] for the treatment of PTSD, which proposes that pathological fear structures need to be activated as completely as possible to enable processing. The stressful event is represented by three-dimensional objects, images, sounds, and music, which can be enriched by the patient with significant personal items such as photographs. Moreover, the therapist has options to modify landscapes and environments in real time (eg, daytime, weather) if he wants to enhance or reflect on the patient’s emotions. Such interactive components are a noteworthy feature of computer-assisted mental health interventions. Results from a preliminary case study point to favorable effects of the 6 week VR supported program with regard to posttraumatic growth, depression, and negative affect [3]. However, the program has not yet been evaluated in a large-scale empirical study.

## Methods

### Own Self-Help Approach

In order to bridge the gap between demands and supply in adjustment disorder interventions, a bibliotherapeutic self-help manual for adjustment disorder was developed [37], based on the diagnostic concept of the ICD-11 [10]. The manual targets the specific group of burglary victims as a first sample case into the field of adjustment disorders. Burglary is experienced as a severe violation or intrusion of the victim’s privacy, possibly resulting in adjustment disorder. Symptoms range from anxiety, anger, and hostility to sadness, for example [38]. Moreover, victims frequently report avoidance of thoughts and feelings related to the event and concern for the future in combination with hypervigilance [39].

In an ongoing project by our team, the unguided self-help manual is implemented as a stand-alone eHealth intervention. The self-help manual in its bibliotherapeutic as well as its Internet presentation follows cognitive-behavioral principles, integrating formerly validated exercises from treatment approaches for PTSD, anxiety disorders, or depression. The exercises were specifically selected to work on symptoms of preoccupation (eg, constant rumination, excessive worry about the stressor) and failure to adapt (eg, difficulties concentrating, sleep disturbance, loss of interest in previously enjoyable activities). The manual is meant to be worked on during approximately 4 weeks, guided by a timetable outlining the course of the treatment,

- In the first part of the manual, a self-test for the screening of adjustment disorder symptoms (Adjustment Disorders - New Module-6) [40] is provided, which helps the reader to evaluate the psychological burden caused by a stressful life event. Subsequently, the individual stress reaction is acknowledged as a normal response to an unusual situation, and it is pointed out that strong feelings are not uncommon in such situations. Sympathy and understanding is shown to further increase the therapy motivation.
- The second part of the manual is predominantly psychoeducational and provides information about the causes of adjustment disorder, its symptoms, and its possible consequences. It also contains a checklist for evaluating if face-to-face contact with a trained mental health professional was more appropriate than the self-help approach.
- The third part constitutes the main body of the self-help manual and comprises the CBT exercises that are structured in four chapters: (1) *Sense of self,* the aim is to guide the person to understand the origin of his or her stress-response, including an analysis of risk and protective factors. Psychoeducation on the mutual influence of feelings and cognitions is provided and an evaluation of previous coping strategies performed. (2) *Coping*, a variety of cognitive strategies such as the recognition and correction of mental biases are presented as well as thought-stopping techniques and strategies for the management of anxieties. As in the treatment of PTSD, a written narrative exposition exercise is suggested. (3) *Activation*, exercises aiming at introducing positive emotions, activation of personal resources, and forming and expressing positive and realistic personal goals. The reader receives information on the advantages of physical activities and is motivated to engage in athletic activities. And (4) *Recovery*, the importance of balancing activity on the one hand and relaxation on the other hand is stressed, and psychoeducation on the physical processes during relaxation is provided. A variety of relaxation exercises and a focus on sleep hygiene complete the intervention. While readers are advised to work through exercises in chapters 1 and 2 successively, exercises in chapters 3 and 4 are advised to be chosen according to personal priority and taste.

The following case description illustrates the course and outcomes of the self-help manual for a person suffering from burglary-triggered adjustment disorder. The sample case stems from an evaluative study of the bibliotherapeutic form of the manual.

### Case Illustration

Mr. H. is a 68-year-old married man who lives together with his wife in a house owned by the family. They have no children and he is retired. Approximately eight months ago, he was victimized in a burglary. During the night, when he and his wife were asleep, the burglars entered his home, and with it his most private space. Although Mr. H. was in his bedroom, the burglars did not hesitate to steal most of his electronic devices and caused financial damage of over 10'000 Swiss Francs (approx. US $10,000). Mr. and Mrs. H. did not hear or see the burglars. They became aware of the situation the next morning when they woke up and called the police immediately.

Mr. H. was horrified over what had happened to him and his wife. He reacted with physical arousal such as sweating, shivering, and heart palpitation. In addition, he felt very angry and frustrated that he could not do more himself. Although the burglars did not vandalize the house, Mr. H. himself felt tainted. Mr. H. has suffered from “psychological pain”, as he called it ever since, despite the fact that his insurance covered the material damage. It was the first time somebody had entered his home without permission, and for him it was very difficult to adapt to this thought. The weeks passed and Mr. H. could not really adapt to the situation. It all did not make sense to him, and he asked himself, “Why me?” “Why now?” “Will it happen again?”. He spent much time ruminating about the burglary and felt that he could not control his thoughts in this respect.

Therefore, he signed up for participation in the study investigating the psychological effects of burglary on the victims and evaluating the above mentioned self-help manual. Although Mr. H. had never worked with a self-help guide before, he was confident that it could help him. During his participation, Mr. H. had to fill out questionnaires at three points in time.

### Course of Study

**Figure 1:**
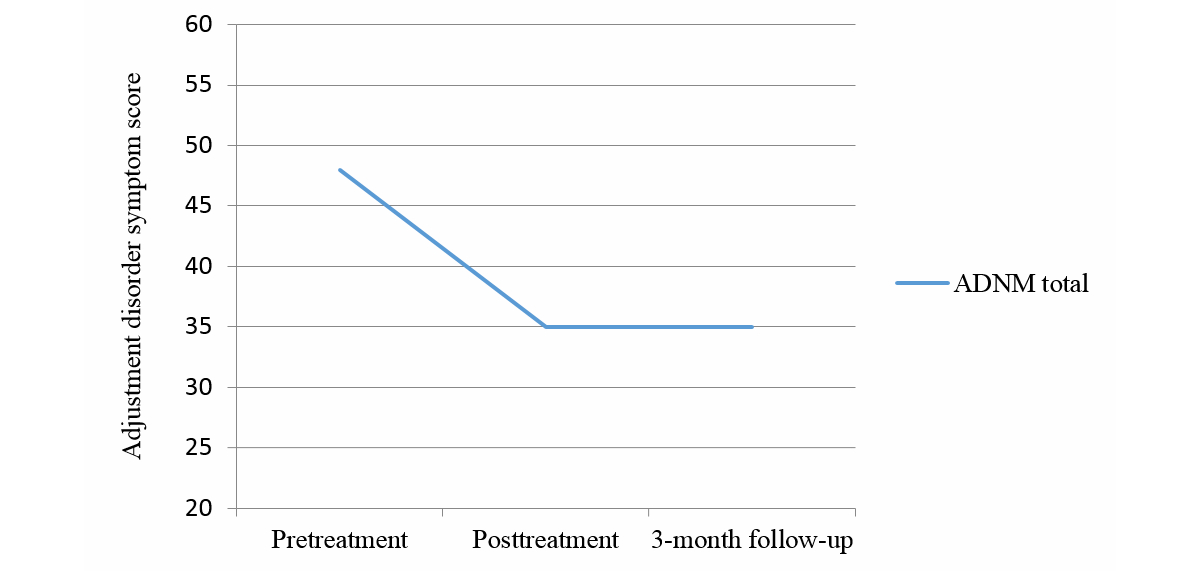
Adjustment Disorder – New Module-20 (ADNM) total symptom improvement (the total score comprises preoccupation, failure-to-adapt, and accessory symptoms).

**Figure 2:**
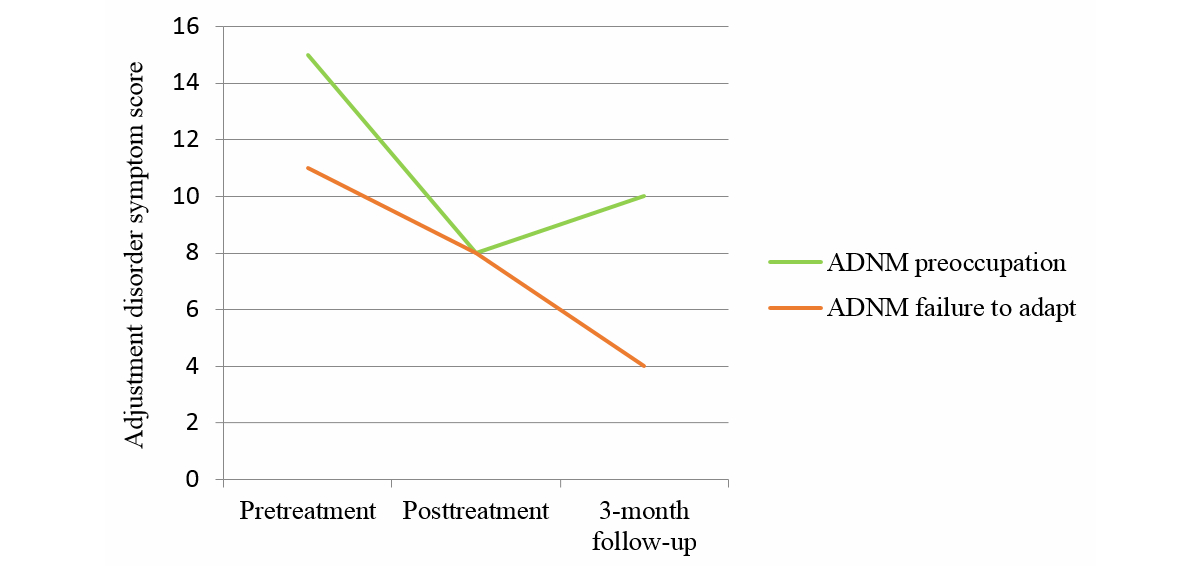
Symptom improvement on subscales of the Adjustment Disorder – New Module-20 (ADNM).

The first survey contained questions about the burglary, and Mr. H.s’ emotional reactions. According to the new ICD-11 concept of adjustment disorder [10], Mr. H fulfilled the criteria of an adjustment disorder with strong symptoms of preoccupation and failure to adapt. In the Adjustment Disorder New Module-20 [41], a questionnaire specially designed for the ICD-11 adjustment disorder, Mr. H reached a total score of 48 (scale range 20-60). He scored 15 (range 4-16) points on the preoccupation subscale and 11 (range 4-16) points on the failure to adapt subscale. Figures 1 and 2 show graphic illustrations of the symptom decrease between pre and posttreatment. On the Depression Anxiety Stress Scales (DASS-21) [42], an established instrument to measure low positive affectivity (depression), physiological arousal (anxiety), and stress, he scored a total of 24 out of 63 points. He reached higher scores on the depression and anxiety subscales than on the stress subscale.

Mr. H. worked with the treatment manual for four weeks. He took out the book several times a week for two or three hours, so that in the end of the fourth week, he had worked on all the exercises in the book. In total, he worked for about 15 hours during the four weeks. He thought that almost all the exercises helped him to deal with his pain over the incident.

## Results

### Second Measurement

At the time of the second measurement, the symptoms of adjustment disorder had decreased for Mr. H., and he did not fulfill the adjustment disorder criteria anymore. He scored a total of 35 points with 8 points on the preoccupation subscale and 8 points on the failure to adapt subscale (Figure 2). Thus, he showed a substantial decrease in preoccupation (7 points) and failure to adapt symptoms (3 points). It seems as if the manual had helped him to learn to manage his thoughts and feelings about the burglary. He still thought about it from time to time, but not as often as before. Moreover, the thoughts did not cause as much pain as before, and it was easier now to adapt to the situation. In the DASS-21, he showed a slight decrease of symptoms on the depression (two points) and anxiety subscales (one point). The measures for the DASS-21 are presented in Table 1.

**Table 1:** Pretreatment and posttreatment measures on DASS-21.

	Pretreatment	Posttreatment	3-month follow-up
DASS-21 total^a^	24	21	16
Depression^b^	4	2	0
Anxiety^c^	4	3	1
Stress^d^	2	2	0

^a^DASS-21 total range 0-63

^b^depression range 0-21

^c^anxiety range 0-21

^d^stress range 0-21

### Three Month Follow-Up Assessment

After three months, Mr. H. participated in the follow-up assessment of the study. He still did not fulfill the criteria of an adjustment disorder. Some symptoms of preoccupation had reoccurred, but apparently he learned how to handle them. Adaption to the situation did not seem to be a problem anymore as is represented by the lowest possible scoring on the failure to adapt subscale. In the long run, the manual seemed to help Mr. H. by showing him ways on how to deal with the difficult thoughts and feelings that occurred when being reminded of the burglary. The depression, anxiety, and the stress symptoms were also reduced during the three months after Mr. H. had worked with the self-help manual.

Overall, the treatment with the self-help manual reduced symptoms of adjustment disorder, namely preoccupation and failure to adapt, as well as symptoms of depression, anxiety, and stress. Mr. H. reports that it was the combination of exercises, rather than an individual exercise, which helped him in dealing with difficult feelings and thoughts related to the burglary. He did not point out any specific exercise that helped the most. Before treatment, he suffered from recurrent thoughts about the loss and problems to adapt to the altered circumstances for eight months. After one month of working on the subject with cognitive behavioral methods as captured in the self-help manual, a significant change in symptom severity was reached. After three months of treatment, symptom severity was still considerably lower than before treatment. Although some preoccupation symptoms recurred, symptoms of failure to adapt declined again. This case demonstrates that a low intensity intervention on the basis of a cognitive behavioral approach, can achieve positive short- and long-term results in the treatment of adjustment disorder.

## Discussion

### eHealth Treatments for Adjustment Disorders

With this paper, we want to show promising prospects for eHealth treatments for adjustment disorder. Adjustment disorder is a highly prevalent, but underresearched, mental disorder that is undergoing significant changes with regard to its diagnostic conceptualization in the course of DSM-5 and ICD-11 revisions.

Discriminant validity is improved by the definition of specific diagnostic criteria, especially with regard to depressive episodes. This development lays the foundation for future research on development and upholding factors as well as intervention methods. Despite the extensive need for interventions, the current empirical evidence for the effectiveness of psychotherapeutic treatment is sparse in the face-to-face context, as well as in e-mental health. Individual studies based on various theoretical backgrounds such as CBT, dynamic approaches, or client-centered psychotherapy are encouraging, but need to be replicated. The domain of e-mental health has only reluctantly begun to expand to adjustment disorder, with one approach demonstrating the usage of VR to complement face-to-face therapeutic contact [3]. A new unguided self-help manual, which is currently adapted to an Internet-based format, shows positive preliminary results, as was illustrated in a case study.

The structure, distribution, and costs of mental health care make it unavailable for a significant part of the world’s population [43]. Taking into account the high occurrence rates of serious nontraumatic stressors and the considerable prevalence rates of adjustment disorder, a continued promotion of eHealth approaches might prove uniquely capable for meeting the diverse demands for scientists, health care practitioners, and patients. From a research perspective, an e-intervention enables the researcher to get information on user behavior (eg, which components are accessed most often) and to evaluate the effectiveness of different modules. Furthermore, it can contribute to reduce the translational lag in transfer of research findings into regular mental health care [44]. From the perspective of health care providers, it could be used to propose a cost-effective prevention measure as soon as a disturbing critical life event gets reported. This way, it could be possible to reduce the chance for subsequent disorders for which adjustment disorder can act as a precursor [45,46]. From the viewpoint of those possibly affected by adjustment disorder, a website can potentially reach out to persons that otherwise would not be able to access mental health care. Even more important, it initially eliminates the stigma connected to seeing a therapist [32], while providing clear guidelines to assess whether professional care is indicated.

Preliminary results from the evaluation study of the new unguided self-help manual for burglary victims with adjustment disorder are encouraging. The manual is one of the few interventions specifically targeting the symptoms of adjustment disorder, and the first one to be based on the revised concept according to ICD-11, to our knowledge. In a case study, symptoms of preoccupation and failure to adapt, as well as symptoms of depression, anxiety, and stress were successfully reduced. This case demonstrates that with low intensity intervention on the basis of a cognitive behavioral approach, positive short- and long-term results in treatment of adjustment disorder can be achieved. CBT interventions are particularly well suited for adaption to a computer format due to their structured, mostly modular organization and the focus on behavior and cognition [47].

A second domain of e-mental health interventions comprises interventions with varying degrees of therapist contact, namely, guided self-help approaches. Such treatments seem promising for the future as their effectiveness was shown for various mental disorders, for example [2]. Even though some advantages of unguided self-help are attenuated, such as cost-effectiveness or accessibility, guided self-help interventions are usually associated with higher adherence to treatment, less dropouts, and higher effects than unguided interventions. Furthermore, the approach by Andreu-Mateu et al [3] that combined VR and face-to-face elements was evaluated positively in two case studies. Its inspiration from PTSD therapy focusing on activation of pathological fear structures makes it a particularly interesting procedure [36]. This is in line with the new conceptualization of adjustment disorder as a stress-response syndrome. More traditionally, however, therapist contact in guided self-help interventions takes place via the Internet, for example, in the form of weekly emails [48]. Such approaches would be desirable for adjustment disorder treatment, but have not yet been identified in the literature.

To date, the Spanish VR study [3], as well as our own self-help approach for treating burglary victims with adjustment disorder [37], have been evaluated only in case studies. We are currently testing the bibliotherapeutic manual in a randomized controlled clinical trial. With regard to the Internet version, a pilot study for its implementation into clinical practice is in preparation. Additionally, the manual is currently constricted to treating the specific group of burglary victims suffering from adjustment disorder symptoms. In the future, the intervention should be adapted to more diverse populations suffering from different stressful life events such as job loss, relationship break-up, or financial problems.

### Conclusions

Present classificatory changes pave the way for further research on conceptual theories of adjustment disorder, upholding factors as well as therapeutic interventions. On the one hand, this concerns interventions in the domain of traditional face-to-face psychotherapy; on the other hand, the sector of e-mental health is encouraged to expand to this currently underrepresented territory. E-mental health options are considered uniquely suited for offering early intervention after the experiences of stressful life events that potentially trigger adjustment disorder.

## Abbreviations

BDI: Beck Depression Inventory
CBT: cognitive behavior therapy
DASS-21: Depression Anxiety Stress Scales
DE: depressive episode
DSM-5: Diagnostic and Statistical Manual of Mental Disorders, 5th Version
EMDR: eye movement desensitization and reprocessing
HPA: hypothalamic-pituitary-adrenocortical
ICD-11: International Classification of Diseases 11
PTSD: posttraumatic stress disorder
VR: virtual reality
